# Effectiveness of virtual teaching programme regarding palliative care on knowledge, self-efficacy and attitude of Nursing Personnel in North India

**DOI:** 10.17533/udea.iee.v42n1e04

**Published:** 2024-04-27

**Authors:** Alka Guleria, Kanika Rai, Yogesh Kumar, Jyoti Sarin

**Affiliations:** 1 MSN, RN, Nursing Tutor, Maharishi Markandeshwar Institute of Nursing, Maharishi Markandeshwar (deemed to be university), Mullana, Ambala, Haryana, India. Maharishi Markandeshwar University Maharishi Markandeshwar Institute of Nursing Maharishi Markandeshwar India; 2 Ph.D, RN. Chitkara School of Health Sciences, Chitkara University, Punjab, India. Email: kanika@chitkara.edu.in, nehukanu@gmail.com (Corresponding author) Chitkara University Chitkara School of Health Sciences Chitkara University Punjab India kanika@chitkara.edu.in; 3 Centre for Evidence Based Practice in Health Care, Chitkara School of Health Sciences, Chitkara University, Punjab, India Chitkara University Centre for Evidence Based Practice in Health Care Chitkara School of Health Sciences Chitkara University Punjab India; 4 Ph.D, RN. Professor, College of Nursing, Teerthankar Mahavir University, Moradabad, Uttar Pradesh, India College of Nursing Teerthankar Mahavir University India; 5 PhD, RN. Principal, Maharishi Markandeshwar College of Nursing, Maharishi Markandeshwar (deemed to be university), Mullana, Ambala, Haryana, India. Maharishi Markandeshwar University Maharishi Markandeshwar College of Nursing Maharishi Markandeshwar India

**Keywords:** palliative care, telemedicine, control groups, Knowledge, self-efficacy, attitude to health, nursing staff, hospital, effectiveness., cuidados paliativos, telemedicina, grupos control, conocimiento, autoeficacia, actitud frente a la salud, personal de enfermería en hospital, efectividad., cuidados paliativos, telemedicina, grupos controle, conhecimento, autoeficácia, atitude frente a saúde, recursos humanos de enfermagem no hospital, efectividad.

## Abstract

**Objective.:**

To evaluate the effectiveness of Virtual Teaching (VT) Programme regarding palliative care on knowledge, self-efficacy and attitude among Nursing Personnel working in selected hospitals of North India.

**Methods.:**

A quasi-experimental study with non-equivalent control group pre-test-post-test design was conducted on 121 Nursing Personnel, selected by convenient sampling technique. Knowledge, self-efficacy and attitude were assessed using structured knowledge questionnaire, Palliative Care Self-efficacy Scale, and Frommelt Attitudes toward care of dying scale respectively. Nursing personnel in experimental group received Virtual Teaching Programme regarding palliative care whereas those in comparison group received conventional teaching (CT). The study included a pre-test followed by the teaching (virtual/ conventional) on day one. The post-test was conducted on 15^th^ day after the intervention.

**Results.:**

The results showed that there was a significant difference in mean post-test knowledge (VT group: 17.11 to CT group: 25.05; t=9.25, *p*<0.001), self-efficacy (VT group: 39.27 to CT group: 43.38; t=6.39, *p*<0.001) and attitude (VT group: 108.86 to CT group: 133.23; t=9.27, *p*<0.001) scores between virtual teaching group and conventional teaching group. ANCOVA test revealed statistically significant differences in the mean scores of knowledge [F (1.11) = 86.61, *p*<0.001], self-efficacy [F (1.11) = 841.75, *p*<0.001] and attitude [F (1.11) = 82.92, *p*<0.001] between the groups, with higher means obtained in the CT group.

**Conclusion:**

Virtual Teaching programme and Conventional teaching both were effective in enhancing the knowledge, self-efficacy and attitude among Nursing Personnel regarding palliative care with conventional teaching being more effective.

## Resumen

**Objetivo.:**

Evaluar la efectividad de un programa de Enseñanza Virtual (EV) sobre cuidados paliativos en cuanto a conocimientos, autoeficacia y actitud entre el personal de enfermería que trabaja en hospitales seleccionados del norte de la India.

**Métodos.:**

Se llevó a cabo un estudio cuasiexperimental con un diseño de grupo de control no equivalente. Se realizaron pre y post-prueba a 121 miembros del personal de enfermería, seleccionados mediante una técnica de muestreo por conveniencia. Se evaluaron los conocimientos, la autoeficacia y las actitudes mediante un cuestionario estructurado de conocimientos, una escala de autoeficacia en cuidados paliativos y una escala de actitudes de Frommelt hacia el cuidado de personas al final de su vida. El personal de enfermería del grupo experimental recibió un programa de EV sobre cuidados paliativos, mientras que el del grupo de comparación recibió enseñanza convencional (EC). El estudio incluyó una preprueba seguida de la enseñanza virtual o convencional el primer día y una prueba posterior al decimoquinto día después de la intervención.

**Resultados:**

. Los resultados mostraron que había una diferencia significativa en las puntuaciones medias post-test entre los grupos en: conocimientos (EV: 17.11 y EC: 25.05; t=9.25, *p*<0.001), autoeficacia (grupo VT: 39.27 y grupo CT: 43.38; t=6.39, *p*<0.001) y actitud (grupo EV: 108.86 y grupo EC: 133.23; t=9.27, *p*=<0.001) La prueba ANCOVA también reveló diferencias estadísticamente significativas en las puntuaciones medias de conocimientos [F (1.11) = 86.61, *p*=<0.001], autoeficacia [F (1.12) =841.75, *p*=<0.001] y actitud [F (1.11) = 82.91, *p*<0.001] entre los grupos, obteniéndose medias más altas en el grupo CT.

**Conclusión.:**

Tanto el programa de enseñanza virtual como la enseñanza convencional fueron efectivos para mejorar los conocimientos, la autoeficacia y la actitud del personal de enfermería en relación con los cuidados paliativos, siendo el beneficio mucho mayor la enseñanza convencional.

## Resumo

**Objetivo.:**

Avaliar a eficácia de um programa de Aprendizagem Virtual (VE) sobre cuidados paliativos em termos de conhecimento, autoeficácia e atitude entre a equipe de enfermagem que trabalha em hospitais selecionados no norte da Índia.

**Métodos.:**

Um estudo quase experimental foi conduzido com um desenho de grupo controle não equivalente. Foram realizados pré e pós-testes em 121 membros da equipe de enfermagem, selecionados por meio de técnica de amostragem por conveniência. Foram utilizados os instrumentos: questionário de conhecimento estruturado, escala de autoeficácia em cuidados paliativos e escala de atitudes de Frommelt em relação ao cuidado de pessoas em fim de vida. A equipe de enfermagem do grupo experimental recebeu um programa de VE sobre cuidados paliativos, enquanto o grupo controle recebeu ensino convencional (CE). O estudo incluiu um pré-teste seguido de ensino virtual ou convencional no primeiro dia e um pós-teste no 15º dia após a intervenção.

**Resultados.:**

Os resultados mostraram que houve diferença significativa nas médias dos escores pós-teste entre os grupos em: conhecimento (EV: 17.11 e EC: 25.05; t=9.25, *p*<0.001), autoeficácia (grupo VT: 39.27 e grupo CT: 43.38; t=6.39, *p*<0.001) e atitude (grupo EV: 108.86 e grupo EC: 133.23; t=9.27, *p*=<0.001). O teste ANCOVA também revelou diferenças estatisticamente significativas nas pontuações médias de conhecimento [F (1.11) = 86.61, *p*=<0.001], autoeficácia [F (1.12) =841.75, *p*=<0.001] e atitude [F (1.11) = 82.91, *p*<0.001] entre os grupos, obtendo maiores médias em o grupo CT.

**Conclusão.:**

Tanto o programa de ensino virtual quanto o ensino convencional foram eficazes na melhoria do conhecimento, da autoeficácia e da atitude da equipe de enfermagem em relação aos cuidados paliativos, sendo o benefício muito maior com o ensino convencional.

## Introduction

Approximately 55 million people die each year in the globe today. With the accelerated ageing process, this number is likely to rise rapidly. Despite an increase in palliative care practitioners and services in many nations, the likelihood of a rise in the number of terminally ill individuals passing away is concerning.^1^Approximately 40 million people annually require palliative care, with 78 percent residing in low- and middle-income nations. Approximately 14% of those who require palliative care worldwide currently have an access to it. Palliative care has been accessible in India, despite its limited availability, for nearly two decades. The urgency of delivering palliative care has changed dramatically in the thoughts of health care clinicians and policymakers during the previous two decades. In India, more than 60 patients die of cancer and suffering every hour. Furthermore, with over billion people distributed across a large geopolitical terrain, palliative care's reach may look unattainable.^2^The results of a survey conducted in the state of Haryana during 2016 revealed that the percentage of deaths due to cancer accounted for about 14.2% of the total deaths among the males and females aged 40 to 69 years.^3^

Palliative care is only available to a fraction of one percent of India's 1.2 billion residents. Pioneering efforts in the latter half of the century resulted in development, some of which can be seen in all developing countries. Kerala, accounting for three percent of India's total population remains at the forefront of palliative care provision.^4^Symptom management, psychosocial and spiritual support, and treatment geared specifically towards the underlying illness are the elements that make up palliative care. There is no single care that is adequate if it is not considered in conjunction with other two.^5^

Taking care of issues other than physical pain is an important component of dealing with suffering. Palliative care offers a team approach to help parents and caregivers. This entails providing practical assistance as well as bereavement counselling. It functions as a support system for patients, enabling them to die as comfortably as feasible.^6^Once nurses are able to identify the patient's symptoms and needs, they have the option of providing effective palliative care.^7^^)^

Knowledge, ideas, beliefs, and skills of health care professionals are one of the most essential elements impacting the efficacy of palliative care administration. These factors determine not only the strategy that health care professionals take during patient evaluation, but also the behaviour that they exhibit while doing so. Since nurses are responsible for the physical, functional, social, and spiritual aspects of care, they are the second-most crucial part of the palliative care team after doctors.^8^

There has been evidence that nurses and other medical professionals are ill-equipped to handle patients who are suffering. Numerous variables have been found, including insufficient knowledge, a lack of coursework on pain treatment, and attitudes and beliefs regarding pain in schools.^9^The barriers affecting the provision of palliative care can be removed or modified by careful assessment and treatment of patients requiring the care. 

Palliative care in Indian setting is still not widely researched area as very few studies were found that were conducted on knowledge, self-efficacy as well as attitude regarding palliative care among Nursing Personnel. Moreover, the literature shows that the nursing personnel particularly in India, are not well prepared for taking care of patients in need of palliative care owing to lack of professional education about the same. Nurses must be educated and trained to provide palliative care to patients who require it. The need of the hour is to make content that not only meets the curriculum but also keeps students interested.^10^ The authors of the present study urged to work on the area which is not readily addressed despite having an importance in clinical nursing. This study sought to assess the effectiveness of a virtual teaching programme on nursing personnel's knowledge, self-efficacy, and attitudes regarding palliative care. 

## Methods

A sample of 122 nursing personnel was enrolled in the study which was carried out during 2021-22. The sample size estimated was 99 based on the projected effect size of 0.39 at a power of 0.80. But due to COVID restrictions, a sample of 122 nursing personnel was only available for data collection. Out of total sample, there was an attrition of one Nursing personnel. The final study's sample size was 121. A "Quasi-Experimental (Non-Equivalent control group pre-test post-test design)" was carefully chosen to proceed with this study. This research included those having smart phones and who were willing to participate. The nursing personnel not available during data collection and not able to attend complete virtual teaching (VT) or conventional teaching (CT) regarding palliative care were not included in the research. The investigation was carried out among nursing personnel working in two tertiary care hospitals established under MMDU trust at Mullana (Haryana) and Solan (Himachal Pradesh). 

Ethical Consideration. The institutional ethical committee of MMDU, Mullana granted ethical approval (IEC-1843) to perform the study. There was no/minimal ethical risk included in the study as per the guidelines of Indian Council of Medical Research Guidelines. The formal permission for conducting final study was taken from MMIMSR&H, Mullana and Medical superintendent of MMMC&H, Solan. The nursing staff gave informed consent after being guaranteed that their responses would be kept confidential. 

**Figure 1:**
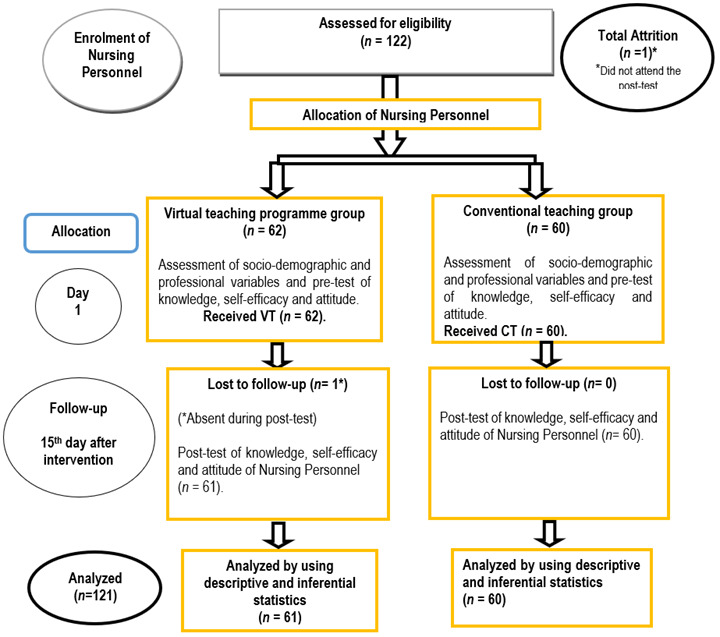
CONSORT Diagram showing flow of sampling

A sample of 62 Nursing Personnel from MMIMSR&H, Mullana and 60 Nursing Personnel from MMMC&H, Solan were conveniently recruited for the final study. On day one, pre-assessment of socio-demographic and professional variables and knowledge, self-efficacy and attitude of Nursing Personnel was done through Google forms using the tools for data collection in VT group and CT group. After taking pre-test, Virtual teaching programme for 1hr 15 min regarding palliative care was administered live to Nursing Personnel in VT group via zoom platform in which case scenarios were also discussed. Whereas conventional teaching regarding palliative care was given for 1hr 15 min via lecture cum discussion method in a group of two having 30 Nursing Personnel each in CT group. The teaching content was same in VT group and CT group and was delivered by researcher. On day 15, post-test of knowledge, self-efficacy and attitude of Nursing Personnel was conducted in VT group and CT group through Google forms. The sampling flow is depicted in Figure 1. 

The tools used for the study were a Structured knowledge questionnaire, Palliative Care Self-Efficacy Scale and FATCOD -Frommelt attitudes toward care of the dying- scale. The structured knowledge questionnaire was formulated with the input of seven research experts and considering prior investigations, with the aim of evaluating the level of knowledge possessed by nursing personnel with respect to palliative care. There were thirty multiple-choice questions containing a single valid response. Each correct response received a point value of one, while any incorrect response received a point value of zero. Therefore, a minimum score of zero and a maximum score of 30 were applicable. A standardized tool was used to assess the self-efficacy regarding palliative care among Nursing Personnel.^11^ It comprised twelve items, each of which was graded on a four-point scale, was positive, and was arranged in direct scale order. A higher score corresponds to an increase in self-efficacy. On a four-point scale, 1 indicates the need for additional fundamental instruction, 2 indicates the ability to perform with close supervision or coaching, 3 indicates the capability to perform with minimal consultation, and 4 indicates the confidence to perform independently. Probable scores cover a range of 12 to 48, with 12 representing the minimum and 48 the maximum. The highest scores indicate greater self-efficacy, while the lowest scores indicate reduced self-efficacy. FATCOD -Frommelt attitudes toward care of the dying- scale was used to assess the attitude of nursing personnel.^12^The assessment comprised 30 Likert-type items, each of which was graded on a five-point scale. Out of the total items, 15 were worded positively (1, 2, 4, 10, 12, 16, 18, 20, 21, 22, 23, 24, 25, 27, and 30) and 15 were worded negatively (3, 5, 6, 7, 8, 9, 11, 13, 14, 15, 17, 19, 26, 28, and 29). Positive items were assigned scores ranging from five (indicating strong agreement) to one (indicating strong disagreement). When evaluating negative objects, the scoring system was reversed. Scores can vary between 30 and 150. Greater ratings indicate more favourable attitudes. The reliability values of all the above-mentioned tools were 0.70, 0.70 and 0.67 respectively. All the standardized tools were incorporated in the study after getting prior permission from the tool developer. The SCVI of structured knowledge questionnaire was 0.84. 

## Intervention

Virtual teaching programme regarding palliative care was developed for nursing personnel to enhance their knowledge, self-efficacy, and attitude regarding palliative care through zoom platform which includes palliative care content, case scenarios, video, audio (experience shared by nursing personnel with terminally ill patients). The areas covered in the teaching plan included concept of palliative care, communication skills, assessment, and management in terminal illness (pain, dyspnoea, constipation, diarrhoea, nausea/vomiting, anorexia, delirium, malignant wound). The duration of teaching was one hour 15 minutes. The virtual teaching was given live via zoom platform that also included the discussion of case scenarios, whereas those in conventional group received the teaching by lecture-cum-discussion. The teaching content was same in both the groups. 

Data were analyzed using SPSS version 20 after checking normality of data by Kolmogorov-Smirnov test. Descriptive analysis was used for the categorical variables like age (in years), gender of the participants, level of education, present area of work, experience in present working area, previous area of work, total working experience. The mean, standard deviation, and median were employed to illustrate the continuous variables. Using the independent t-test, continuous variables were compared. 

## Results

Since the Data was normally distributed, parametric tests were applied for the analysis. Less than half of the nursing personnel (42.6%) and more than half (63.3%) were in the age group 25-28 years in VT group and CT group. All the nursing personnel were female (100%). More than half of the nursing personnel (68.9%), (66.7%) were having level of education of GNM in VT group and CT group respectively. More than half of the nursing personnel's (57.4%), (76.7%) present area of work was General ward in VT group and CT group respectively. More than half of the nursing personnel (70.5%), (63.3%) had ≤ 3 years of total working experience. Most of the nursing staff in both groups (91.8%) and (96.7%) had not received any training in palliative care. The two groups were comparable in terms of personal and professional characteristics except age, present area of work and total work experience. (Table 1) 

**Table 1:** Comparison of VT group and CT group in terms of socio-demographic and professional variables of the nursing personnel regarding palliative care

Socio-demographic and professional variables	**VT Group (*n* = 61) f (%)**	**CT Group (*n* = 60) f (%)**	_*X*_ 2	DF	***p-v*alue**
Age (in years)			9.23	3	0.02
21-24	22 (36.1)	19 (31.7)			
25-28	26 (42.6)	38 (63.3)			
29-32	10 (16.4)	3 (5)			
33-36	3 (4.9)	0			
Gender			--	--	--
Female	61 (100)	60 (100)			
Level of education			0.68	2	0.71
GNM	42 (68.9)	40 (66.7)			
B.Sc. Nursing	10 (16.4)	13 (21.7)			
Post Basic B.Sc. Nursing	9 (14.8)	7 (11.7)			
Present area of work			21.65	4	<0.001
General ward	35 (57.4)	46 (76.7)			
ICU/CCU	17 (27.9)	7 (11.7)			
Emergency/OT	0	7 (11.7)			
Onco/ radiotherapy ward	2 (3.3)	0			
OPD/ MRD	7 (11.5)	0			
Experience in present working area			3.22	1	0.07
Less than 5 years	52(85.2)	57(95)			
5-10 years	9(14.8)	3(5)			
Total working experience			12.89	3	<0.001
< 3	43(70.5)	38(63.3)			
4-6	7(11.5)	20(33.3)			
7-9	10(16.4)	2(3.3)			
10-12	1(1.6)	0			
Do you know about palliative care?			2.27	1	0.13
Yes	54(88.5)	47(78.3)			
No	7(11.5)	13(21.7)			
If yes, source of information:			8.43	4	0.07
Curriculum	7(13)	11(23.4)			
Clinical Exposure	27(50)	23(48.9)			
Internet source	4(7.4)	8(17)			
In-service programme	10(18.5)	2(4.3)			
Any other	6(11.1)	3(6.4)			

The present study revealed a significant distinction between the VT and CT group in terms of knowledge, self-efficacy and attitude of Nursing Personnel regarding palliative care with conventional teaching being more effective. (Table 2) 

**Table 2:** Comparison of VT group and CT group in terms of post-test knowledge, self-efficacy and attitude of Nursing Personnel

Variables	MEAN			t-value	***p*-value**
	VT group (*n* = 61)	CT group (*n* = 60)		
Knowledge	17.11 ± 5.27	25.05 ± 4.07	9.25	<0.001
Self-efficacy	39.27± 4.07	43.38± 2.88	6.39	<0.001
Attitude	108.86± 14.74	133.23± 14.14	9.27	<0.001

The mean pre-test and post-test scores of knowledge, self-efficacy and attitude of Nursing Personnel in the VT and CT groups were compared using paired t-test and significant differences wars found in knowledge, self-efficacy and attitude. In both groups improved scores were observed in in both groups compared the first and second time of measurement. 

To further elucidate this difference, a one-way ANCOVA was conducted to compare the groups whilst controlling for the pre-test scores. There was a significant difference in the mean knowledge [F(1.11) = 86.61, *p*<0.001], self-efficacy [F(1.11) = 841.75 *p*<0.001] and attitude [F(1.11) = 82.91, *p*<0.001] between the groups. (Table 4-a) The comparison of the estimated marginal means showed that there was an improvement in knowledge, self-efficacy and attitude in the CT group (mean: 25.043, 43.420 & 133.206 respectively) as compared to VT group (mean: 17.122, 39.242 & 108.896 respectively). (Table 4-b). 

**Table 3:** Comparison of VT group and CT group in terms of knowledge, self-efficacy and attitude of Nursing Personnel before & after administration of VTP and CT

		Mean		t value	p-value
		Pre test	Post test		
VT Group (*n* = 61)	Knowledge	12.31 ± 3.63	17.11 ± 5.27	6.66	<0.001
	Self-efficacy	33.19± 6.49	39.28± 4.07	6.13	<0.001
	Attitude	96.60± 9.77	108.86±14.74	5.41	<0.001
CT Group (*n* = 60)	Knowledge	12.40 ± 5.12	25.05 ± 4.07	15.62	<0.001
	Self-efficacy	33.58± 6.21	43.38± 2.88	9.34	<0.001
	Attitude	93.53± 10.70	133.23± 14.14	17.02	<0.001

**Table 4-a:** Tests of Between-Subjects Effects in terms of Knowledge, Self-Efficacy and Attitude of Nursing Personnel

Dependent Variable: Post-Knowledge			
Type III Sum of			Partial Eta
Squares	df Mean Square F	***p*-value**	Squared
1897.447	1 1897.44 86.61	<0.001	0.423
2584.988	118 21.90		
Dependent Variable: Post-Self Efficacy			
520.974	1 520.974 41.75	<0.001	0.261
1472.361	118 12.478		
Dependent Variable: Post-Attitude			
17455.100	1 17455.10 82.91	<0.001	0.413
24840.047	118 210.50		

**Table 4-b:** Estimated Marginal Means of Knowledge, Self-Efficacy and Attitude of Nursing Personnel

Group	Mean Post Knowledge	Std. error	Pre-Knowledge
VT Group	17.12	0.59	12.36
CT Group	25.04	0.60	
	Mean Post Self-efficacy	Std. error	Mean Pre - Self- efficacy
VT Group	39.24	0.45	33.84
CT Group	43.42	0.45	
	Mean Post Attitude	Std. error	Mean Pre- Attitude
VT Group	108.89	1.86	95.12
CT Group	133.20	1.88	

## Discussion

After VTP and CT, there was a significant rise in knowledge scores in the present investigation from in VT and CT group. There is some evidence to suggest that these findings are similar, at least in part, with the findings of the study that was carried out by Yanping Hao *et al*. ^13^in which PCQN's total score improved from 10.3±1.9 to 11.1±2.2 (*p*=0.011), while pain and other symptom management's score escalated from 7.7±1.7 to 8.7±1.7 (*p*=0.003). Identical results were reported by Joy^14^ where following participation in an educational intervention, nurses in the intervention group had a discernible 12 percentage point increase in their level of knowledge on palliative care.^14^^)^

In the present study, both VTP and CT were effective but CT was more effective as compared to VTP. In a study that was carried out by Boxel van Patris *et al.*^15^ on the usefulness of videoconferencing in comparison to face-to-face palliative care teaching, the researchers came to conflicting conclusions. The nurses reported high levels of satisfaction with the instructional presentation, regardless of the mode in which it was delivered. It's possible that the time limits of the workshop could have been responsible for the ineffectiveness of videoconferencing when it came to psychological or emotional topics. 

In the present study, VT group and CT group showed an increase of mean self-efficacy scores following the VTP and CT in VT group and CT group respectively. A similar study that was carried out by Phillips *et al*.^11^ found a 6.5-point rise in palliative care post-test scores among RNs after a multimodal intervention, with a post-test score of 38.7. This gain accounted for 13.5 percent of the total increase. These findings were inconsistent with the findings reported by Jin Sun Kim *et al.* ^16^ which had a mean score of 33.8/48 for palliative care self-efficacy. The nurses lacked trust in their abilities to communicate with patients who were in their final stages of life and the relatives of such patients, as well as handling delirium. 

The findings of the present study have implications in the field of nursing education and practice. The nursing students shall study about the end-of-life experience, pain and symptom management, care objectives, and early care planning. Also, in-service education programmes regarding palliative care may be organized for the nursing staff to prepare them for taking care of the terminally ill patients. 

Conclusion. The findings of this study demonstrated that Virtual teaching programme and Conventional teaching both were effective with Conventional teaching being more effective than Virtual teaching programme in enhancing knowledge, self-efficacy and attitude among Nursing Personnel regarding palliative care. Hence, organizing educational programme regarding palliative care for in-job nursing personnel is recommended to prepare them for a competent delivery of palliative care to patients. 
